# Immunodominant HIV-1 Cd4+ T Cell Epitopes in Chronic Untreated Clade C HIV-1 Infection

**DOI:** 10.1371/journal.pone.0005013

**Published:** 2009-04-07

**Authors:** Danni Ramduth, Cheryl L. Day, Christina F. Thobakgale, Nompumelelo P. Mkhwanazi, Chantal de Pierres, Sharon Reddy, Mary van der Stok, Zenele Mncube, Kriebashne Nair, Eshia S. Moodley, Daniel E. Kaufmann, Hendrik Streeck, Hoosen M. Coovadia, Photini Kiepiela, Philip J. R. Goulder, Bruce D. Walker

**Affiliations:** 1 HIV Pathogenesis Programme, Doris Duke Medical Research Institute, Nelson R. Mandela School of Medicine, University of KwaZulu-Natal, Durban, South Africa; 2 Ragon Institute of MGH, MIT and Harvard, Massachusetts General Hospital East, Charlestown, Massachusetts, United States of America; 3 Department of Pediatrics, Nuffield Department of Medicine, The Peter Medawar Building for Pathogen Research, Oxford University, Oxford, United Kingdom; 4 Howard Hughes Medical Institute, Chevy Chase, Maryland, United States of America; New York University School of Medicine, United States of America

## Abstract

**Background:**

A dominance of Gag-specific CD8+ T cell responses is significantly associated with a lower viral load in individuals with chronic, untreated clade C human immunodeficiency virus type 1 (HIV-1) infection. This association has not been investigated in terms of Gag-specific CD4+ T cell responses, nor have clade C HIV-1–specific CD4+ T cell epitopes, likely a vital component of an effective global HIV-1 vaccine, been identified.

**Methodology/Principal Findings:**

Intracellular cytokine staining was conducted on 373 subjects with chronic, untreated clade C infection to assess interferon-gamma (IFN-γ) responses by CD4+ T cells to pooled Gag peptides and to determine their association with viral load and CD4 count. Gag-specific IFN-γ–producing CD4+ T cell responses were detected in 261/373 (70%) subjects, with the Gag responders having a significantly lower viral load and higher CD4 count than those with no detectable Gag response (p<0.0001 for both parameters). To identify individual peptides targeted by HIV-1–specific CD4+ T cells, separate ELISPOT screening was conducted on CD8-depleted PBMCs from 32 chronically infected untreated subjects, using pools of overlapping peptides that spanned the entire HIV-1 clade C consensus sequence, and reconfirmed by flow cytometry to be CD4+ mediated. The ELISPOT screening identified 33 CD4+ peptides targeted by 18/32 patients (56%), with 27 of the 33 peptides located in the Gag region. Although the breadth of the CD4+ responses correlated inversely with viral load (p = 0.015), the magnitude of the response was not significantly associated with viral load.

**Conclusions/Significance:**

These data indicate that in chronic untreated clade C HIV-1 infection, IFN-γ–secreting Gag-specific CD4+ T cell responses are immunodominant, directed at multiple distinct epitopes, and associated with viral control.

## Introduction

HIV-1 infection is characterized by a loss of CD4+ T cells. In particular activated HIV-1-specific memory CD4+ T cells are preferentially infected and progressively depleted from both the gastrointestinal associated lymphoid tissue (GALT) and the periphery [1], [2]. This depletion of the target cells at mucosal sites is mirrored in the pathogenic simian immunodeficiency virus (SIV) infection of rhesus macaques [3], [4]. Studies of chronic infections in animal models and humans including HIV-1, hepatitis C virus, malaria, and even bacterial infections have demonstrated that optimal CD8+ T cell activity is dependent on CD4+ T cells [1]–[4]. However, the relationship between HIV-1 clade C virus infection and HIV-specific T helper cell function has not been examined.

Previous studies have suggested that the preservation of HIV-1 specific CD4+ T cell responses might be critical for the control of viral replication [5]–[7]. In subjects with long-term non-progressive HIV-1 infection HIV-1-specific CD4+ T cell responses are typically present; in contrast, they are progressively lost in subjects with progressive infection and high levels of viral replication. Moreover, subjects infected with HIV-2, which is characterized by a less malignant disease course, generally exhibit strong virus-specific CD4+ T cell responses with the ability to simultaneously secrete multiple cytokines [8]. In both cases it has been suggested that the preservation of HIV-1-specific CD4+ T cell responses might be a crucial component in the overall immune responses in maintaining control over viral replication.

Apart from the decline in CD4+ T cell numbers, HIV-1 infection also impairs the functional and phenotypic heterogeneity of HIV-1 specific CD4+ T cells. In untreated HIV-1 clade B infection, characterized by antigen persistence and high antigen load, HIV-1-specific CD4+ T cell responses tend to be weak or absent. Detectable virus-specific CD4+ T cell cytokine responses in HIV-1 infection consist mainly of IFN-γ, whereas in subjects able to control viral replication CD4+ T cells that secrete IL-2 are also detectable, and may be a key component of an effective immune response. Central memory T cells produce mainly IL-2 while effector memory cells produce both IFN-γ and IL-2 [9]. Persistent exposure to antigen, such as occurs in HIV-1 infection, is believed to generate short-lived IFN-γ producing effector memory CD4+ T cells which are impaired in their ability to develop into IL-2 producing central memory cells [10]. This phenomenon has been observed at all stages of the infection and is postulated to disrupt the IL-2 producing capacity of CD4+ T cells directed against HIV-1 [11].

This skewing of cytokine secretion is in turn believed to diminish the ability of these cells to proliferate in response to HIV-1 antigens [12]. Very early studies of HIV-1 specific CD4+ T cell responses found strong proliferative responses against the p24 Gag antigen in individuals who were able to control HIV replication without therapy [6]. However many studies have shown that chronic progressive infection is associated with no or minimal proliferative responses while IFN-γ producing HIV-1 specific CD4+ T cells are retained [5], [13]–[16]. Taken in the context of IL-2 producing and memory T cells, the resultant concept is that persistent HIV-1 antigenemia skews the production of functional IL-2 producing central memory CD4+ T cells to a less functional IFN-γ producing phenotype that reduces the proliferative capacity of HIV-1 specific CD4+ T cells [10].

The vast majority of studies that have contributed to our current understanding of CD4+ T cell immunology in HIV-1 infection have been conducted in the context of clade B infection, which is the predominant clade in North America and Europe. The brunt of the AIDS epidemic is however borne by Sub-Saharan Africa where clade C virus predominates. In 2007 alone, 32% of all new HIV infections globally occurred in Southern Africa [17]. Understanding the immunology of HIV-1 specific T cell responses in Southern Africa is therefore imperative in designing a vaccine for this particular region but there have been limited analyses of HIV-1 specific CD4+ T cell responses in the context of clade C HIV infection [18], [19]. The identification of HIV-1 CD4+ T cell epitopes and an understanding of the relationship between these responses and viral control is crucial in designing an HIV-1 vaccine that elicits protective CD4+ T cell responses. Neither the elucidation of these epitopes, nor the pattern of cytokine production by HIV-1 specific CD4+ T cells has been determined.

Here we evaluate the hypothesis that HIV-1-speccific CD4+ T cell responses to clade C virus infection are associated with control of viremia, by analyzing a large cohort of persons with chronic untreated clade C virus infection. We demonstrate that the HIV Gag protein is the major target for HIV-1-specific CD4 T cell responses in clade C virus infection, and show that Gag-specific CD4+ T cell responses in chronically infected clade C subjects are significantly associated with lower viral load and higher CD4 counts. In addition, we identify HIV-1 clade C CD4+ T cell epitopes and find that the majority of these epitopes are located in the Gag region. These data demonstrate an immunodominance of Gag-specific CD4+ T cell responses related to control of viremia in clade C virus infection.

## Results

### IFN-γ–producing, Gag-specific CD4+ T cell responses are associated with lower viremia and higher CD4 counts

**Figure 1:**
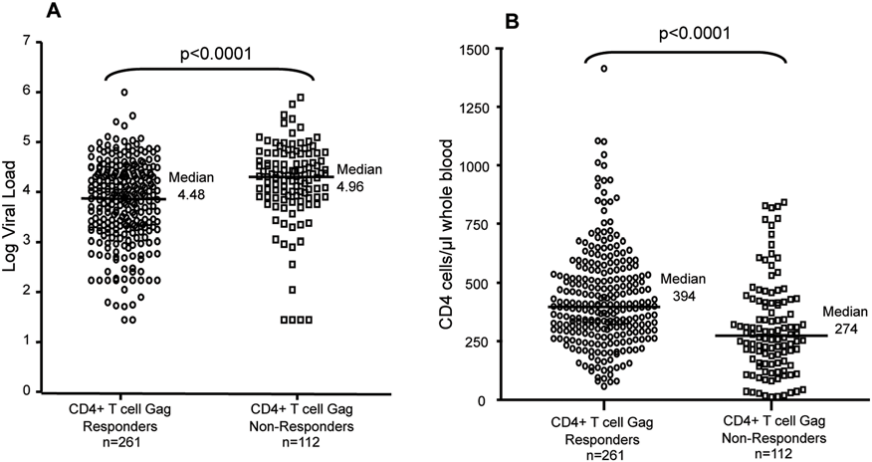
CD4+ T cell responses targeting HIV Gag are associated with immunological control. Persons with a CD4+ T cell response against a pool of Gag peptides have significantly lower viral loads and higher CD4 counts than those with no response against the Gag peptide pool. A total of 373 subjects were screened and statistics were calculated using the Mann Whitney test.

Previous studies in clade B virus infection have suggested that robust HIV-1-specific CD4+ responses are mounted during primary HIV-1 infection, but subsequently lost during disease progression, whereas the preservation of detectable Gag-specific CD4+ T cell responses has been associated with a slower disease progression [20]. We were therefore interested to assess, in a large untreated cohort, whether the presence of Gag-specific CD4 responses is associated with the level of viremia. PBMCs from 373 patients with chronic, untreated clade C infection were stimulated with a pool of HIV-1 clade C Gag peptides and screened for IFN-γ-producing CD4+ T cell responses by intracellular cytokine staining (ICS) assay. Among the 373 subjects tested for Gag-specific CD4+ T cell 70% (261/273) showed detectable IFN-γ secretion upon antigenic stimulation. Interestingly, the subjects with the highest IFN-γ CD4+ T cell response appeared to also have the lowest viral loads. We next compared the subjects with a detectable Gag-specific CD4+ response with subjects who lacked significant IFN-γ secretion upon Gag stimulation. The 261 subjects with a detectable CD4+ T cell response had a median viral load of 29,900 RNA copies/ml plasma [range 49 to 8.5×10^6^] which was significantly lower than the median viral load of subjects without a detectable CD4 response (median 91,900 RNA copies/ml plasma) [range 49 to 6.5×10^6^] (p<0.0001, Mann Whitney, Figure 1A), although there was considerable overlap. The 112 subjects without a detectable CD4+ T cell response also had a significantly lower CD4+ T cell count (p<0.0001, Mann Whitney, Figure 1B, median CD4 count 274 vs 394 of Gag responders). As it has been demonstrated that HIV-1 preferentially infects HIV-1-specific CD4+ T cells [21], it is plausible that the lack of CD4+ T cell responses might also reflect the stage of disease progression. The distribution of the data showed that some patients with a positive CD4+ Gag-specific response had viral loads greater than 100,000 RNA copies/ml, while 4 patients with no CD4+ Gag specific response had undetectable viral loads (<50 copies/ml plasma). Thus, although the presence of a CD4+ Gag specific response is significantly associated with a low viral load and higher CD4+ T cell count, it does not always predict a state of viral control.

### The HIV-1 Gag protein has the highest CD4+ T cell epitope density

**Figure 2:**
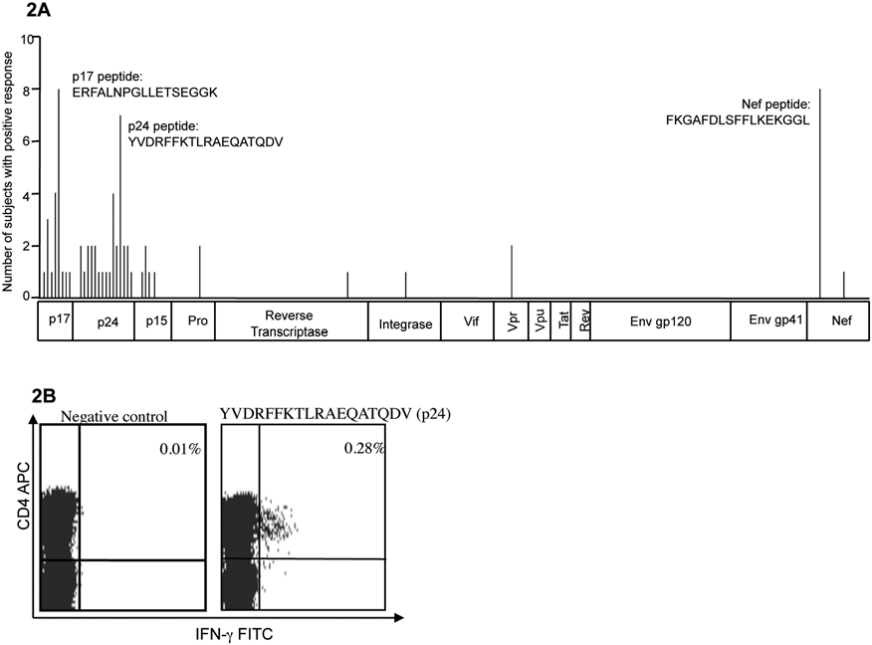
The majority of the HIV-1 C clade CD4+ epitopes cluster in the Gag region. ELISPOT screening was conducted on 32 randomly selected subjects. (A) 33 CD4+ restricted epitopes were identified, with 27 epitopes located in the Gag region, 3 in the Polymerase region, 1 in the Vpr region and 2 in the Nef region. There were no CD4+ epitopes present in the Vif, Vpu, Rev, Tat, or Envelope proteins. (B) The peptides identified by the ELISPOT assay utilizing CD8+ depleted PBMCs were confirmed by flow cytometry using whole PBMCs to be CD4+ restricted. The dot plot above represents data from the negative control on the left and a CD4+ IFN-γ response to the p24 Gag peptide YVDRFFKTLRAEQATADV.

Previous studies in clade B infection as well as limited studies in clade C infection have shown the HIV-1 Gag protein to be the dominant target of virus-specific CD4+ T cell responses [18], [20]. However, little is known regarding the exact regions of the HIV proteome that are preferentially targeted by HIV-1-specific CD4+ T cell responses in clade C infection. In order to define the target antigens and the relative immunodominance of the overall HIV-1-specific CD4 T cell response in clade C virus infection, we used an IFN-γ Elispot assay to screen for HIV-1-specific CD4+ responses using an overlapping peptide set spanning the entire HIV-1 clade C proteome in 32 randomly selected subjects. CD8-depleted PBMC were used in the Elispot assay to identify specific peptides eliciting a CD4+ T cell response. A total of 33 peptides containing CD4+ T cell epitopes were identified, with 27 peptides located in the Gag region, 13 of which were targeted by two or more persons (Figure 2A). The most commonly targeted CD4+ peptides were a p17 Gag peptide (ERFALNPGLLETSEGGK) and a Nef peptide (FKGAFDLSFFLKEKGGL), each targeted in 8 of the 32 subjects, followed by a p24 Gag peptide detected in 7 of the subjects (YVDRFFKTLRAEQATQDV, Table 1). There was a paucity of peptides containing epitopes in HIV-1 proteins other than Gag: 3 in the Polymerase region (protease; reverse transcriptase and integrase), 2 in Nef, 1 in the Vpr region and none in the Vif, Vpu, Rev, Tat or Envelope proteins. Each peptide identified was confirmed to be CD4+ restricted by flow cytometric analysis of PBMCs (representative data Figure 2B).

**Table 1:** Most frequently recognized clade C CD4+ T cell epitopes.

Protein	Sequence	Number of subjects with response	% Of Subjects with response	Range of response (SFCs/million CD8 depleted cells)
p17	ERFALNPGLLETSEGCK	8	25	70–870
p17	GKKHYMLKHLVWASREL	3	9	120–320
p17	ASRELERFALNPGLL	4	12.5	70–700
p24	QMVHQAISPRTLNAWVKV	2	6	70–120
p24	YVDRFFKTLRAEQATQDV	7	22	70–700
p24	AFSPEVIPMFTALSEGA	2	6	170–470
p24	GGHQAAMQMLKDTINEEA	2	6	110–240
p24	LHPVHAGPIAPGQMREPR	2	6	90–840
p24	PVGDIYKRWIILGLNKIV	4	12.5	60–650
p24	WIILGLNKIVRMYSPVSI	2	6	75–680
p24	DVKNWMTDTLLVQNA	2	6	65–220
p24	MTDTLLVQNANPDCKTIL	2	6	150–540
P15	GKIWPSHKGRPGNFLQSR	2	6	800–940
Protease	FIKVRQYDQIPIEICGKK	2	6	60–110
Vpr	SRIGILRQRRARNGASRS	2	6	90–210
Nef	FKGAFDLSFFLKEKGGL	8	25	70–850

**Figure 3:**
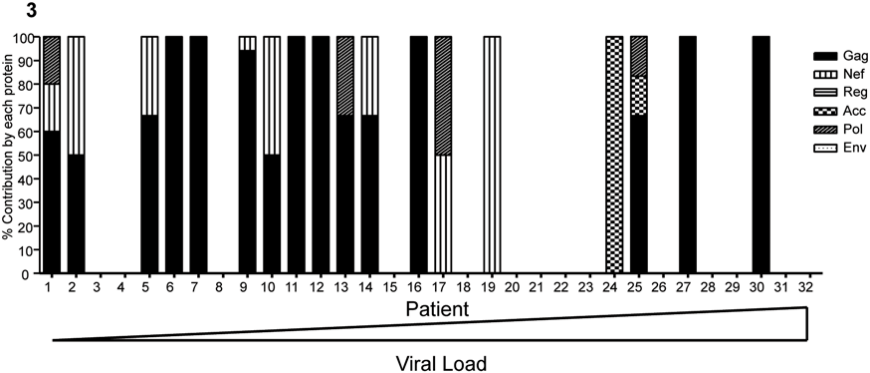
The total HIV-1–specific CD4+ T cell response is dominated by Gag. Using IFN-γ ELISPOT assay to identify HIV-1 specific CD4+ T cell epitopes in 32 subjects, we found that irrespective of the viral load, the Gag protein is the most commonly targeted and therefore makes the greatest contribution to the overall CD4+ T cell response. Subjects 1–16 had viral loads below the median, while subjects 18–32 had viremia greater than the median (36 550 copies/ml plasma). Acc (accessory) denotes the Vpr, Vpu and Vif proteins pooled together. Subjects 24 and 25 both targeted a single Vpr protein.

Eighteen of the 32 screened subjects (56%) had detectable HIV-1-specific CD4+ T cell responses, with fifteen of these subjects targeting the Gag region (Figure 3). When Gag was targeted it always comprised at least 50% of the total HIV-1-specific CD4 T cell response, indicating a relative immunodominance of this antigen. Unlike clade B infection, in which Gag and Nef CD4+ T cell responses dominated, we noted a restriction of CD4+ T cell responses to the Gag protein. This difference in the detection of Nef responses could be due to the different treatment status of our subjects compared to those of the clade B study [20].

### The breadth of CD4+ responses correlates inversely with viral load

**Figure 4:**
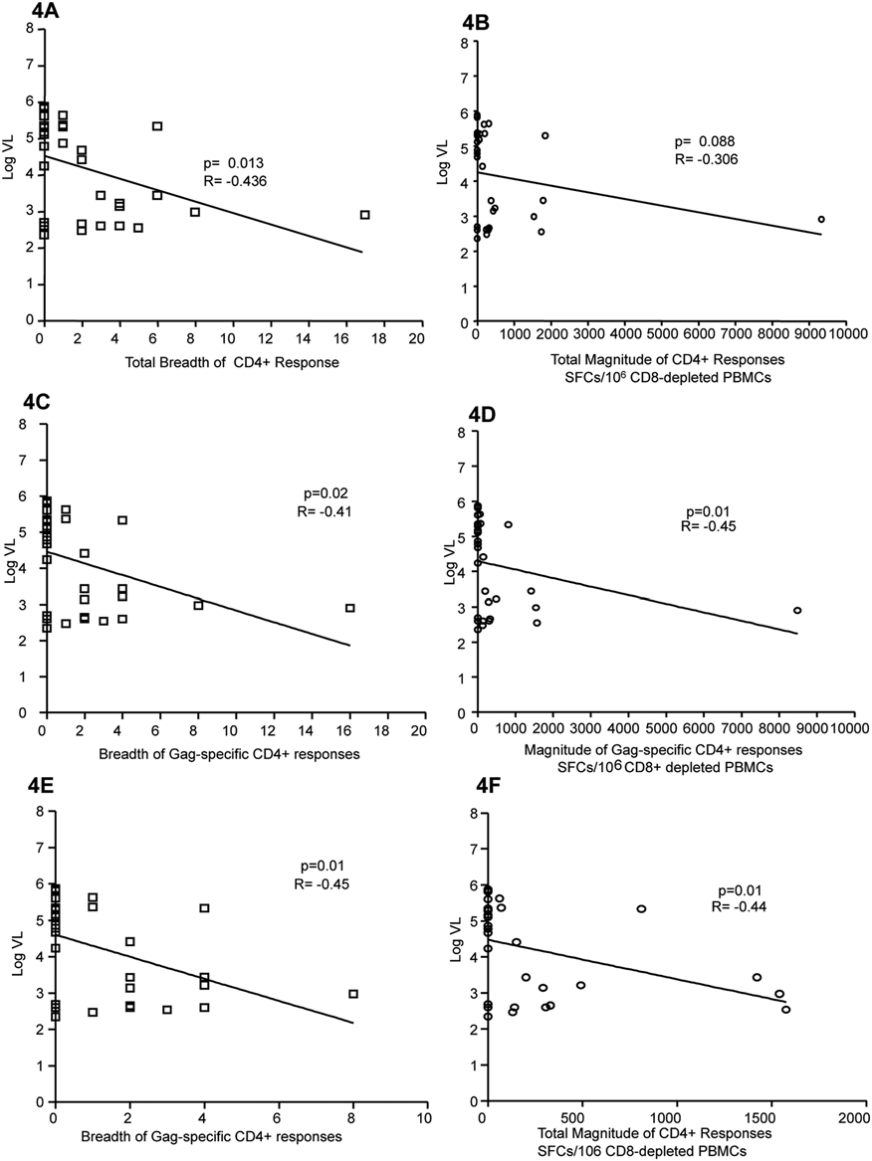
Correlation of total breadth and magnitude of CD4+ T cell response to viral load. (A) The total breadth of the HIV-1-specific CD4+ T cell response was obtained by summing the number of peptides identified by the ELISPOT assay and was found to correlate inversely with viral load. (B) The total magnitude of the response was calculated by adding the number of spot forming cells for each peptide response. Here we found no association between the magnitude of the response and viral load. More specifically the breadth (C) and magnitude (D) of Gag-specific CD4+ T cell responses, correlated inversely with viral load. Excluding the outlier with 17 peptide responses, these correlations continued to be significant for both breadth (E) and magnitude (F).

As it has been recently suggested that the breadth of HIV-1-specific CD8+ T cell responses is significantly associated with disease progression in HIV-1 clade C infection [22], we were interested in whether this is also the case for HIV-1-specific CD4+ T cell responses. The total breadth of the CD4+ T cell response was obtained by summing the number of peptides identified by ELISPOT assay. While the total magnitude of the response was not significantly associated with the viral load level (p = 0.088, R = −0.306, Spearman Rank correlation, Figure 4B), we found a significant inverse correlation between the breadth of response and viral load (p = 0.013, R = −0.436, Spearman Rank, Figure 4A). When looking more specifically at the breadth and magnitude of Gag-specific CD4+ T cell responses, we found an inverse correlation between each of these parameters and viral load (p = 0.02, R = −0.41 for breadth and p = 0.01, R = −0.45, Spearman Rank correlation; Figure 4C and 4D). These correlations continued to be significant even when the single patient with the highest number and magnitude of Gag-specific responses was excluded (p = 0.01 for breadth and magnitude vs VL, Figure 4E and 4F). These data suggest that the number of peptides targeted may be particularly important in viral control. Similar analyses in larger cohorts is required to investigate if the induction of a broad range of CD4+ T cell responses induced by HIV-1 peptides are protective, and to what extent the breadth of responses to proteins other than Gag may contribute to control.

### IFN-γ producing HIV-1–specific CD4+ T cells dominate over IFN-γ/IL-2 HIV-1–specific CD4+ T cells

**Figure 5:**
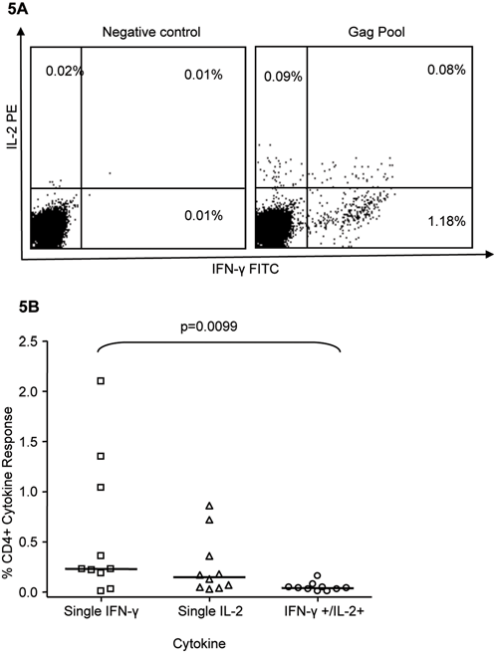
Dual cytokine staining to pools of overlapping peptides. Dual IFN-γ/IL-2 ICS was conducted on 10 subjects. (A) The dot plot on the left shows the unstimulated control while the one on the right shows the simultaneous detection of IFN-γ and IL-2 in response to stimulation with a pool of Gag peptides. The cells were gated on CD4+ T cells. (B) The cytokine response to all HIV-1 protein pools were added to obtain the total HIV-1 cytokine specific response. The percentage of HIV-1-specific CD4+ T cells producing only IFN-γ was significantly higher than the percentage of HIV-1-specific CD4+ T cells producing both IFN-γ and IL-2.

Previous studies found an inverse correlation between HIV-1-specific CD4+ T cells producing IL-2 or IFN-γ/IL-2 and viremia [23], [24]. In order to further characterize the HIV-1-specific CD4+ T cell responses in chronic clade C infection; we stimulated PBMCs from 10 chronically infected patients with pooled HIV-1 synthetic peptides spanning all HIV proteins and assayed for the simultaneous production of IFN-γ and IL-2 by CD4+ T cells by ICS and flow cytometry. Representative data are provided in Figure 5A, where distinct populations of IFN-γ-single positive, IL-2-single-positive, and IFN-γ/IL-2-double positive CD4+ T cells are seen. The responses to all HIV-1 protein pools were then added together to represent the total HIV-1 cytokine specific response. We found that the percentage of HIV-1-specific CD4+ T cells producing IFN-γ only was significantly higher than the percentage of HIV-1 specific CD4+ T cells simultaneously producing both IFN-γ and IL-2 (p = 0.0099, Kruskal-Wallis analysis and corrected for multiple comparisons with Dunns Multiple Comparison Test, Figure 5B). These data indicate that, similar to clade B infected subjects, there is a lack of IL-2 production by HIV-1-specific CD4+ T cell responses associated with chronic infection and chronic exposure to antigen [11], [14], [25]. Unlike previous publications, we found no correlation between Gag-specific IL-2 and total IL-2 production and viral load (p = 0.13, R = −0.52, Spearman Rank, data not shown). This could be due to the small number of subjects evaluated for multiple cytokine secretion, and further studies are clearly warranted.

### High degree of cross clade recognition for the most commonly targeted p24 Gag peptide

Our data above indicate a preference for CD4+ T cells to target epitopes within the Gag HIV-1 protein. We next sought to determine whether the most immunodominant peptides in clade C and clade B infection are cross-recognized. The 8 most commonly targeted CD4+ T cell epitopes in B clade infection [20] were tested for cross recognition in twenty eight randomly selected patients. Only 3/8 B clade CD4+ T cell epitopes were recognized by just 9/28 individuals of this clade C infected cohort. These peptides were all located in the Gag region: ERFAVNPGLLETSEGCR in p17 Gag, YVDRFYKTLRAEQASQEV in p24 Gag and RQANFLGKIWPSHKGR in p15 Gag. One patient responded to the p15 peptide only, one to the p17 peptide only; five to the p24 peptide and 2 patients recognized both the p17 and p24 peptides.

**Figure 6:**
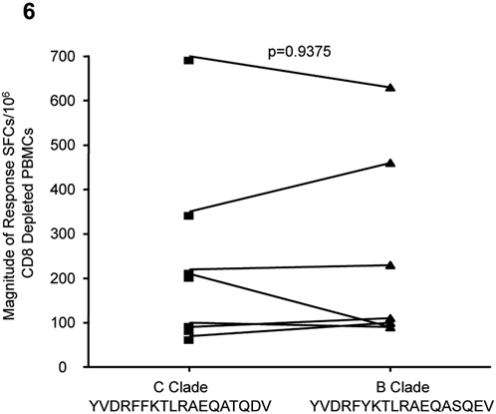
Cross clade recognition of the p24 Gag peptide YVDRFFKTLRAEQATQDV. In a subset of 28 subjects, we found that the C and B clade versions of peptide YVDRFFKTLRAEQATQDV induced the same magnitude of IFN-γ response in 7 patients, as detected by the ELISPOT assay.

We next compared the magnitudes of the clade B and C peptide responses of these 3 peptides to further define the degree of cross-clade recognition of these targeted CD4+ peptides. The clade B version of the p15 peptide, RQANFLGKIWPSHKGR, elicited a response of 70 SFCs while the clade C peptide had a response of 160 SFCs in the same patient (data not shown). The clade B sequence ERFAVNPGLLETSEGCR was recognized by only 3 of the clade C virus infected subjects, all of whom had lower magnitude responses (data not shown). In contrast, all 7 subjects who responded to YVDRFFKTLRAEQATQDV, also responded with similar magnitude to the B clade sequence of the peptide (YVDRFYKTLRAEQASQEV, p = 0.9375, Wilcoxon matched pairs test, Figure 6). These data suggest that cross-clade recognition by HIV-1-specific CD4+ T epitopes is confined to a single p24 Gag peptide, and even minor sequence changes between clade B and clade C might be sufficient to account for lack of cross recognition.

## Discussion

Evidence from both Hepatitis C virus (HCV), HIV-1 and SIV infection suggests that virus-specific CD4+ T cells are crucial in the effective immune control of chronic viral infections [7], [26], [27]. There is a paucity of data on clade C HIV-1-specific CD4+ T cell responses, despite the fact that clade C infection accounts for the majority of infections globally [17], [18]. In this study, we comprehensively assessed HIV-1 specific CD4+ T cell activity in an African cohort of untreated subjects, defining the dominant targeted epitopes, the ability to produce IFN-γ and IL-2, and cross-clade epitope recognition. Our results indicate that 70% of clade C infected persons target the HIV-1 Gag protein which contains the majority of CD4+ T cell epitopes, that targeting of Gag is associated with lower viral loads, and that there is an inverse correlation between the breadth of the HIV-1-specific CD4+ T cell response and viral load.

In an earlier study of 65 chronically infected subjects using clade C peptide pools in an intracellular cytokine staining assay, we found Gag to be the dominant target of HIV-1 specific CD4+ T cells, but could not detect a relationship between the detection of Gag-specific responses and viral load [18]. Here we do find a significant albeit weak association between Gag-specific CD4+ T cell responses and viral load. However, despite the dominance of Gag-specific CD4+ T cell responses and their correlation with lower viral loads, the vast majority of untreated chronically infected patients do progress to AIDS. A possible reason is that these are immunodominant responses captured at a particular timepoint and which wane with continuing viral replication. A longitudinal study that tracks these responses will be able to address this particular point. Proliferative capacity and IL-2 production by HIV-1 specific CD4+ T cells appear to be clearer markers of CD4+ T cell activity than IFN-γ as there is a distinct decline of these parameters in patients exposed to increasing levels of viremia [28]. Although IFN-γ production may also be affected, the decline may be more subtle which could explain why a cross sectional analysis is unable to establish a strong association.

It has been recently demonstrated that the upregulation of inhibitory immunoregulatory molecules, CTLA-4 and PD-1, on HIV-1 specific CD4+ T cells is associated with disease progression [29], [30]. Although not investigated in this study, the PD-1 expression on CD4+ T cells has been shown to be associated with viral load in clade B virus infection [29]. Although it is likely that patients with strong Gag-specific CD4+ T cell responses express fewer CTLA-4 and PD-1 molecules than patients with no Gag-specific CD4+ T cell responses, an additional study will be required to investigate this hypothesis.

Since CD4+ T cells play such a central role in coordinating functions of the immune system, a comprehensive analysis of cytotoxic CD8+ T cell function, neutralizing antibody activity and CD4+ T cell receptor signaling pathways will shed more light on mechanisms of viral control.

The results from our current study using the ELISPOT assay add to our earlier data by demonstrating that the Gag protein contains the highest CD4+ T cell epitope density. Although we only detected 2 epitopes in Nef, one was among the two most highly targeted of all epitopes. Gag and Nef, were also found to be the most frequently targeted proteins by CD4+ T cells in clade B HIV-1 infection and in the less pathogenic HIV-2 infection [20], [31]. The frequent targeting of Gag and the contrasting lack of responses to Vif, Vpu, Rev, Tat and envelope also correlate with observations from clade B CD4+ T cell epitope mapping [20].

The reason for the frequent targeting of Gag by CD4+ T cells and the link with control of viremia, could be associated with the high number of Gag molecules (1500 molecules of Gag and 100 of the precursor Gag-Pol molecules in a single immature virus particle), coupled with a relatively high degree of conservancy of this protein [32], [33]. Among the Gag epitopes targeted, particularly noticeable is the dominant targeting of the p24 Gag peptide YVDRFFKTLRAEQATQDV. This was found to be frequently targeted in clade B and clade A/G HIV-1 infection [20], [31], and displayed a high degree of cross recognition between HIV-1 and HIV-2 infected individuals [31]. Our data have also confirmed that at least for some epitopes, clade C infected individuals can mount an IFN-γ CD4+ T cell response of equal magnitude against both the clade B and C sequences. This peptide overlaps partially with the Major Homology Region (MHR), located at the C terminal dimerization domain of p24 (the capsid antigen) and displays significant homology amongst the various genre of retroviruses [32]. Mutations to the MHR result in viral particles that are defective in viral assembly, maturation and infectivity [32]. The peptide, YVDRFFKTLRAEQATQDV, therefore presents as a possible valuable component in an HIV vaccine and deserves further characterization with regards to the immune response it elicits.

The relative lack of responses in proteins other than Gag may also be due to the use of the consensus clade C sequence which could potentially limit our detection of antigen specific CD4+ T cell epitopes to proteins that are more variable. There was a significant augmentation in the detection of CD8+ T cell epitopes in clade B infection using autologous HIV-1 peptides in comparison to the consensus sequence [34]. This may also be one of the reasons why we detected fewer Nef responses in contrast to the clade B CD4+ epitope screening study [20]. Another reason is that our study focused on chronically infected untreated patients, compromising both controllers and non-controllers of HIV-1 infection, while the clade B study consisted of patients undergoing structured treatment interruption and acutely infected patients. These differing disease states and levels of virus exposure could also account for the differences observed with regard to the Nef responses.

In this study we were able to identify HIV-1 specific epitopes producing IFN-γ only. Since IL-2 and IFN-γ/IL-2 CD4+ T cell responses are associated with control of viremia [12], [23], [24], it will be important to focus future studies on identification the breadth and specificities of clade C IL-2 and IFN-γ/IL-2 producing CD4+ epitopes. Here we observed comparatively sparse IL-2 and IFN-γ/IL-2 HIV-1 specific CD4+ T cells by ICS in our cohort. This could be explained by our cohort being chronically infected, with an immune response already skewed to a dominant IFN-γ only producing effector memory phenotype [10]. A limitation of this study is that because of constraints on the number of parameters available for flow cytometric analysis, we were not able to characterize the polyfunctional capacity and memory phenotype of epitope specific CD4+ T cells. This aspect, along with evaluating CD4+ T cell activity in acutely infected patients, also requires further investigation.

Although the breadth of the CD4+ T cell responses in our cohort correlated inversely with viral load, the magnitude had no significant association with viremia. Research on clade B HIV-1 specific CD4+ T cell responses has revealed that although CD4+ T cell responses can be readily detected, they are generally of low magnitudes [15], [20]. The reason for this observation is unclear but it could be associated with the level of sensitivity of current assays, or to the inability to test with peptides representing autologous viruses. The impact of host genetic factors on HIV-1 specific CD4+ T cell responses in clade C HIV-1 infection also warrants investigation as different HLA Class I molecules have shown to significantly influence CD8+ T cell responses [35]. Analysis of clade B CD4+ T cell epitopes binding to HLA Class II molecules has revealed a high degree of promiscuity amongst the epitopes [20], [36]. This property may also be beneficial in vaccine design as these epitopes can induce responses amongst a wide array of HLA Class II types [36].

In conclusion, dominant HIV-1-specific CD4+ responses are detectable against a limited number of epitopes in chronic HIV-1 clade C infection. Most CD4+ T cell responses are directed against epitopes in Gag and the presence of Gag-specific CD4+ T cells is significantly associated with lower viral load and higher CD4+ T cell counts compared to subjects with no detectable Gag-specific CD4+ T cell responses. Further studies are required to determine whether HIV-1-specific CD4+ T cell activity contributes directly to the control of viremia or is a consequence of viral control. The further identification and characterization of HIV-1 CD4+ epitopes will be important in characterizing clade C immunogenicity and will likely play an important role for clade specific vaccine development.

## Materials and Methods

### Ethics statement

Ethical approval was granted by the Institutional Review Board of Massachusetts General Hospital and the Ethics Committee of the University of KwaZulu-Natal. All participants enrolled in the study signed a written consent form agreeing to participate in the study.

### Study population

Three hundred seventy three subjects, recruited from the Sinikithemba Care Center at McCord Hospital in Durban South Africa, were screened for Gag-specific IFN-γ CD4+ T cell responses by intracellular cytokine staining (ICS) using freshly isolated PBMCs. All subjects were chronically infected with clade C HIV-1 and had not received any antiretroviral medication before or at the time of analysis [median viral load (VL) – 44,300, range 49 to 8.5×10^6^ RNA copies/ml plasma; and median CD4 count 357, range 11 to 1414 cells//µl whole blood]. Thirty two were randomly selected (to exclude the possibility of introducing a bias in our analysis) and screened by an IFN-γ ELISPOT assay (detailed below) using individual HIV peptides to identify CD4+ epitopes. The median viral load of this group was 36,550 RNA copies/ml plasma and median CD4 count 342 cells/µl whole blood. From this group, 10 were randomly selected to assess HIV-1-specific intracellular IFN-γ and IL-2 production by flow cytometry. Twenty eight patients were tested for cross clade recognition of the most common CD4+ epitopes in clade B infection using the IFN-γ ELISPOT assay.

Ten HIV-1 negative subjects (as determined by a negative ELISA and viral load measurement) served as controls.

### CD4+ counts and viral load

Absolute CD4 counts were measured by using the MultiTest TruCount kit and the MultiSet software on the FACS Calibur (Becton Dickinson). Plasma HIV-1 viral loads were measured using the Roche Amplicor kit version 1.5 on the Roche Cobas Amplicor instrument. The sensitivity of the test was 50 RNA copies/ml plasma.

### Intracellular cytokine staining

The intracellular cytokine assay was conducted using peripheral blood mononuclear cells (PBMC) isolated from whole blood within 4 hours of phlebotomy, as described previously [41]. This protocol was followed to assess Gag-specific responses in 373 patients. Changes to the protocol to evaluate IFN-γ and IL-2 cytokine levels included a 12 hour incubation period with pooled and individual peptides; 2 tubes of negative/unstimulated controls per patient (with the average background being subtracted from the experimental tubes). The following antibodies were used: anti-human CD8 Peridinin-chlorophyll-protein Complex (PerCP), anti-human CD4 Allophycocyanin (APC), anti-human IFN-γ Fluorescein isothiocyanate (FITC) and anti-human IL-2 Phycoerythrin (PE).

Ten HIV-1 unexposed, RT-PCR and ELISA negative adults served as controls, where the range of response to any pool of HIV-1 peptides was 0.00–0.04% of CD4+ T cells. A response was therefore considered positive for HIV-1-specific CD4+ T cells if after subtraction of the average background, the response was still greater than 0.04%.

### CD8+ T cell depletion and ELISPOT

Whole blood obtained within 4 hours of phlebotomy was depleted of CD8+ T cells by incubation with anti-CD8 RosetteSep antibody (Stem Cell Technologies) prior to PBMC separation by ficoll-histopaque density centrifugation. The purity of the isolated cells was confirmed by flow cytometry and contained on average 98% CD4+ cells. These cells were used for the ELISPOT assay as described previously [37].

Ninety six-well polyvinylidene difluoride-backed Elispot plates (MAIP S45, Millipore) were coated overnight at 4°C with 100 µl anti-IFN-γ antibody (1-D1k, 0.5 µg/ml, MabTech, Sweden). The plates were then washed six times with blocking buffer (1% fetal calf serum (FCS) in PBS). 50 µl of R10 (RPMI 1640 medium supplemented with 10% FCS, 1% L-glutamine, and 1% penicillin/streptomycin) were added to the empty wells. Ten microliters of pooled overlapping peptides (6 peptides/pool) spanning the entire clade C consensus sequence were added into the wells so that the final concentration of each peptide in a pool was 2 µg/ml in the well. In addition, cells were added to 6 wells containing medium only as a negative control, and 2 wells containing phytohemagglutinin (PHA) (1 µg/ml in the well) as a positive control. Cells were plated at 100 000 cells/well and incubated for 16 hrs at 37°C and 5% CO_2_. The plates were then washed 6 times with PBS and 0.5 µg/ml of biotinylated anti-IFN-γ antibody (7-B6-1, MabTech) was added to the plates which were incubated at room temperature in the dark. The plates were washed once again with PBS and 0.5 µg/ml of streptavidin-alkaline phosphatase conjugated antibody (Mabtech) was added and left in the dark for 45 minutes at room temperature. After a final wash, IFN-γ producing cells were identified by direct visualization of spots produced by the addition of alkaline phosphatase colour reagents (Bio-Rad).

The number of IFN-γ producing cells was quantified by counting the number of spots using an automated ELISPOT plate reader (AID ELISPOT Reader System, Auoimmun Diagnstika, Germany). Results are expressed as the number of Spot Forming Cells (SFCs)/million CD8 depleted PBMCs. A response was considered positive if it exceeded 100 SFCs/million CD8 depleted PBMCs (the highest response observed in HIV-1 negative control patients) and was ≥3 standard deviations above the mean of the 4 negative control wells. Individual peptides identified using the above matrix screenings were reconfirmed by ELISPOT and then by intracellular cytokine staining of PBMCs to be CD4+ restricted.

Eight dominant CD4+ B clade epitopes [20] were individually screened by ELISPOT for cross clade recognition at the same concentration and using the same number of cells as described above. Positive responses were confirmed by ICS assay. The sequences of the 8 peptides screened were: p17 amino acid position 37–51 ASRELERFAVNPGLL; p17 amino acid position 42–58 ERFAVNPGLLETSEGCR; p17 amino acid position 77–94 SLYNTVATLYCVHQRIEV; p15 amino acid position 66–81 RQANFLGKIWPSHKGR; p24 amino acid position 133–150 WIILGLNKIVRMYSPTSI; p24 amino acid position 164–181 YVDRFYKTLRAEQASQEV; Nef PEKEVLVWKFDSRLAFHH amino acid position 176–196; Nef KFDSRLAFHHMARELH amino acid 184–199.

### Statistical analysis

All statistical analyses and graphs were plotted using GraphPad Prism software (version 4). The data were assessed for Gaussian distributions and the relevant statistical test was conducted by the software. A p value of <0.05 was considered significant.
